# Pharmacological Effects of Biosynthesis Silver Nanoparticles Utilizing *Calotropis procera* Leaf Extracts on *Plasmodium berghei*-Infected Liver in Experiment Mice

**DOI:** 10.2147/IJN.S490119

**Published:** 2024-12-21

**Authors:** Mutee Murshed, Jameel AL-Tamimi, Mohammed M Mares, Waleed A Q Hailan, Khalid Elfaki Ibrahim, Saleh Al-Quraishy

**Affiliations:** 1 Department of Zoology, College of Science, King Saud University, Riyadh, 11451, Kingdom of Saudi Arabia

**Keywords:** *Plasmodium berghei*, histopathological, antioxidant, oxidative, cytokine

## Abstract

**Introduction:**

Malaria caused by *Plasmodium* spp. is the most hazardous disease in the world. It is regarded as a life-threatening hematological disorder caused by parasites transferred to humans by the bite of Anopheles mosquitoes.

**Purpose:**

*Calotropis procera* leaf extract combined with biosynthesized silver nanoparticles (CPLEAgNPs) to evaluate its antiplasmodium and hepatoprotective effects against *P. berghei*-induced infection in experimental mice.

**Methods:**

The animal groups were divided into four groups: the first non-infected group was orally administered distilled water daily 7 days. The second group received an oral dose of 50 mg/kg of CPLE AgNPs. The third group received intraperitoneal injections of 10^5^ *P. berghei*. The fourth group received of 10^5^ *P. berghei* with 50 mg/kg CPLE AgNPs. All mice were anesthetized with CO_2_ and dissected for sample collection.

**Results:**

This study of *C. procera* leaves showed that they contain chemically active substances, as shown by the amounts of phenols, flavonoids, and tannins. The antioxidant activity of the samples was assessed using 1.1-diphenyl-2-picrylhydrazyl (DPPH) and 2.2′-azino-bis-3-ethylbenzthiazoline-6-sulphonic acid (ABTS) assays. Treatment of infected mice with CPLE AgNPs for 7 days resulted in a significant decrease in parasitemia and a reduction in histopathological alterations in the liver. Furthermore, CPLE AgNPs mitigated oxidative damage caused by *P. berghei* infection in the liver. In addition, after receiving the medication, the liver levels of alanine aminotransferase, aspartate aminotransferase, and alkaline phosphatase decreased. In addition, CPLE AgNPs regulated the expression of liver cytokines, including IL-1β, and I-10.

**Discussion:**

Based on these findings, the study proved that CPLE AgNPs have hepatoprotective and antiplasmodial properties.

## Introduction

Malaria remains a major public health issue in tropical nations and is one of the most dangerous infectious diseases for which there is no safe and efficient antimalarial vaccine.^1^,^2^ The World Health Organization (WHO) estimated 219 million malaria infections and 435,000 deaths worldwide in 2017, with 61% of deaths occurring in young under five.^3^ A protozoan parasite of the genus Plasmodium causes malaria by growing inside host erythrocytes. This parasite causes both morbidity and mortality in humans. *P. berghei* is a useful rodent model for investigating the role of the liver in blood-stage malarial infection. This is because they share some characteristics with the human pathogen *P. falciparum*.^4^

The enormous effectiveness of medicines (chloroquine and others) and their widespread use in recent years have resulted in resistance to chloroquine in *P. falciparum* and *P. vivax*, the two parasitic species responsible for the majority of human malaria infections.^5^ Medicinal herbs have been used to treat malaria since ancient times.^6^

Despite the phenomenal growth of modern medicine, synthetic drugs available for hepatic disorders are ineffective. Medicinal plants have been used to treat malaria since ancient times. These plants are interesting sources of novel antimalarial drugs.^7^ *Calotropis procera* is a perennial shrub with softwood. It is a member of the family Apocynaceae and subfamily Asclepiadaceae. This evergreen xerophytic plant exhibits a high level of success under dry and semiarid conditions. Various parts of the world refer to this plant by popular names, such as the apple of Sodom, Calotrope, wild cotton, Indian milkweed, huge milkweed, and rubber tree.^8^ However, Saudi Arabia refers to it as “Ushar”. Throughout history, it has been used in traditional medical applications in North Africa, the Middle East, South Asia, and Southeast Asia. Since ancient times, it has been used as fuel, fiber, feed, and lumber since ancient times.^9^ A study^10^ claims that it possesses beneficial activity in the treatment of hepatic disorders. Flavonoids, alkaloids, cardiac glycosides, tannins, sterols, and triterpenes are present in this plant.^11^ Reports have shown that flowers possess anti-inflammatory, antipyretic, analgesic, antimicrobial, and larvicidal properties.^7^ The biosynthesis of metal nanoparticles (NPs) is a burgeoning research topic because of their potential applications in nanomedicine.^12^ Plant extracts can enhance the benefits of biosynthetic NPs by controlling their size, shape, and dispersion.^12^ Recent years have seen a rise in the significance of plant-mediated biological production of NPs has gained significance in recent years because it is both straightforward and environmentally friendly.^13^ The production of AgNPs utilizing natural green sources has numerous advantages. This means that they are non-toxic to animal cells and tissues.^14^ A previous study^15^ demonstrated the significant antiplasmodial action of AgNPs biosynthesized from the aqueous leaf extracts of Saraca indica and Azadirachta indica against *P. falciparum*. Only a handful of experimental treatments for malaria have attempted to use nanocarriers via active targeting techniques. According to,^16^ the liver is the principal regulator that is responsible for the formation of the immune response. In addition to erythrophagocytosis, which is the liver’s important role in iron recycling, the liver also eliminates damaged erythrocytes and potential pathogens such as Plasmodium. The purpose of this research was to investigate whether or not biosynthesized silver nanoparticles made with *C. procera* leaf extracts have an antiplasmodial and hepatoprotective effect on murine blood-stage malaria infection.

## Materials and Methods

### Phytochemical Preparation

*C. procera* leaves were obtained from wild plants in the Riyadh desert (°24′00″N 46°43′00″E / 24.4°N 46.71667°E / 24.4; 46.71667). The collected leaves were validated at the King Saud University Herbarium, (KSU NO:24531), Identified by prof. Mohamed A. El-Sheikh and Dr. Rajakrishnan R. After washing and air-drying, 200 g of the leaves were ground into a powder. Methanolic (80% concentration) was used to extract the powdered leaf components.^2^ Briefly, powdered leaves were agitated in methanol for 48 h. The extract was filtered through (Whatman No. 1) filter paper. The biosynthesized AgNPs were prepared using 5 mL of filtrate and the remaining portion was evaporated using a vacuum evaporator (Yamato RE300, Japan). Distilled water was used to dissolve the powder and kept at −20°C until use.

### Gas Chromatography Mass Spectrometry (GC-MS) Method

Agilent Technologies’ GC-MS 7890B (Santa Clara, CA, USA) autosampler injection system was used. The sample components were identified using GC-MS. A DB-5 MS capillary column from Agilent Technologies (30 m length x 0.25 mm internal diameter, phase thickness 0.25 μm) was employed for the separation of target compounds, utilizing helium as the carrier gas at a flow rate of 1 mL/min. The inlet temperature was set at 250°C with a split ratio of 50, and the oven temperature was programmed from 50 to 250°C, resulting in a total analysis duration of 71 minutes. The MS detector parameters were configured as follows: acquisition scan type, mass range of 40 to 500 g/mol, scan speed of 1.56, a solvent delay of 2 minutes, and an MS source temperature of 230°C.

### Total Polyphenol Content (TPC), Total Flavonoid Content (TFC), and Total Tannin Content (TTC) Measurements

The approach of Behbahani et al was used to calculate the TPC and TFC of the extract. A mixture was produced with 2 mL of distilled water, 100 µL of Folin-Ciocalteu reagent, and 20 µL of extract (concentration: 10 g/L). The mixture was agitated for two hours after adding 300 µL sodium bicarbonate solution for three minutes. The absorbance of the samples was measured at 765 nm using a spectrophotometer (Sigma3, 30k). Gallic acid (0–500 mg/L) was used to generate the standard curve. The TPC was expressed as mg gallic acid (GAE)/g extract according to Behbahani et al.^17^

1mL of the crude extract concentration and 1mL of 2% methanolic aluminum chloride and the mixture was incubated for 15 min at room temperature in the dark. The absorbance values of the samples were measured at 430 nm to determine the TFC. A standard curve was created using quercetin and the TFC value was expressed as μg quercetin (QE) equivalent per gram of dry weight of the extract.^17^ Each 200 mL series of leaf extracts was mixed with 200 mL of Folin-Ciocalteu reagent to determine the tannin concentration, which was then allowed to stand for five minutes. Subsequently, 100 cc of saturated NO_2_CO_3_ and 5 cc of distilled water were added. The absorbance was measured at 745 nm after 35 min of incubation. Tannic acid was used as the standard. The amount of tannin found in each sample was measured in mg of tannic acid equivalent (mg TAE/g d.w.) per gram of dried weight according to Kumar et al.^18^

### Radical Scavenging Method for Antioxidant Activity

Antioxidant activity of the extracts was assessed using a previously described method outlined by.^19^ The extract was diluted to a range of 10–500 μg/mL after the methanolic solution was prepared at a concentration of 1 mg/mL. 1mL of each extract was added to a solution of 1mL methanol and 2 mg DPPH. The absorbance of the extract and blank samples reaction components without extract was measured at 517 nm after the samples were left in the dark for 30 min at 24 °C. The following activity was determined using the antioxidant formula:

$${\mathrm{I\%=}}{{\mathrm{A}}_{{\mathrm{blank}}}}{\mathrm{-}}{{\mathrm{A}}_{{\mathrm{sample}}}}{\mathrm{/}}{{\mathrm{A}}_{{\mathrm{blank}}}}{\mathrm{\times100}}$$

Where A sample and A blank denote the absorptions of the extract and blank samples, respectively. Using the IC_50_ value,^19^ the antioxidant activity of the extract was compared to that of the synthetic and natural antioxidants vitamin C and tert-butylhydroquinone (TBHQ). The slope equation of the radical scavenging activity (RSA) curve was used to determine the IC_50_ value, which is the concentration of the sample that can block 50% of free radicals.

### ABTS Test for Scavenging Free Radicals and Cations

ABTS radical inhibition efficacy of the extract was assessed in.^20^ TBHQ and vitamin C were used as the controls. A solution of free radicals (7 mm ABTS, 2.4 mm potassium persulfate) was prepared and incubated at 24°C for 14 hours in the dark. After adding 200 µL (10–500 μg/mL) of the extracted sample, the free-radical solution (2 mL) was thoroughly mixed. Following a half-hour, the absorbance of the samples was measured at 734 nm, and the ABTS free radical scavenging activity was reported using IC_50_ according to Labiad et al.^20^

### AgNP Biosynthesis and Characterization

The nanoparticles were prepared using a previously described technique according to Anandalakshmi et al.^21^ Briefly, IME (5 mL) was mixed with 8 × 10^3^ M silver nitrate in 45 mL methanol and maintained at 50°C for 60 min until the mixture turned dark brown, suggesting the formation of AgNPs in the solution. The reduced AgNP solution was monitored using UV-visible spectroscopy.

According to Jiang et al,^22^ the form and size of AgNPs were determined using a JEOL JEM-2100 transmission electron microscope (JEOL Ltd., Tokyo, Japan). Briefly, a carbon-coated copper grid was immersed twice in the nanoparticle solution and then placed in an Eppendorf tube. Finally, the copper grid was removed and dried for 4 h at 25 °C for additional morphological analysis.

### Design of an Experiment

The study was carried out in compliance with the “Guide for the Care and Use of Laboratory Animals”. The study adhered to the institutional regulations on animal use at King Saud University and conformed to the criteria established by the National Committee of Bio-Ethics (NCBE) in Saudi Arabia. According to the Royal Decree, bearing the number M59, was issued on 14/9/1431H. The Research Ethics Committee of King Saud University (Approval No. KSU-Se-21-78) approved all the procedures of those experiments, ensuring animal rights, welfare, minimal stress, and no harm or suffering.

Female C57BL/6 mice (9–11 weeks old). The animals were acclimatized to the laboratory circumstances two weeks before the commencement of the experiment and were then kept in stainless steel wire cages (5 animals per cage) under pathogen-free conditions. Animals were given food and water. The temperature was maintained at (23 ± 2 °C) with a relative humidity of (50–60%) and a light/dark cycle of 12 hours each.

To identify the best dose effect for AgNPs, we experimented in the following groups: 5 mice per group: group 1, the infected group by intraperitoneally with 1×105 P. berghei as the control; group 2 (infected + 10 mg/kg CPLE AgNPs); group 3 (infected + 25 mg/kg CPLE AgNPs); and group 4 (infected + 50 mg/kg CPLE AgNPs).

As demonstrated by Wunderlich et al,^23^ cryopreserved blood stages of *P. berghei* were passed through mice and administered intraperitoneally to the experimental animals. A Neubauer chamber was used to calculate the number of 10^5^ parasitized erythrocytes. Blood smears stained with Giemsa were obtained from mouse tails to determine parasitemia and the peak point at which animals should be anatomically collected.^23^ The mice were separated into five groups of 5 mice each. First control group. The 1–5 groups were intraperitoneally infected with 1×10^5^ *P. berghei* –infected erythrocytes as previously described by Fairlie-Clarke.^24^

The experiment was replicated with six mice in each group to investigate the effect of CPLE AgNPs: Group 1, control non-infected control; Group 2, non-infected treated with AgNPs, 50 mg/kg; Group 3, infected group; and Group 4, infected group + CPLE AgNPs, 50 mg/kg. All animals underwent anatomical CO_2_ asphyxiation on day 7 p.i. followed by sample collection.

The change in liver weight was calculated and the liver index was calculated as the ratio of liver weight to mouse weight.

### Counting White Blood Cells (WBCs)

The number of WBCs in the blood of the mice was calculated using a VET-530 CA Medonic blood counter (Stockholm, Sweden).

### Samples Collection

On the 7-day post-infection, all mice were sedated by CO_2_ asphyxiation and then euthanized for the collection of blood and liver tissues. Blood was extracted from the heart and stored in tubes containing EDTA. The blood was divided into two components: one for whole blood and the other for plasma. The plasma was thereafter preserved at −20°C until required. The liver was excised and divided into three segments for histological examination. Samples were kept in neutral buffered formalin (10%) for histological examination. Small pieces of liver were preserved at −80°C for the examination of liver function. The last parts were preserved in RNAlater for molecular investigation of gene expression.

### Hematological Studies

Blood was drawn into heparin-containing tubes (AIN MEDICARE SDN.BHD). 16100 Kota Bharu, Kelantan, Malaysia) to determine key hematological parameters such as erythrocyte count, total hemoglobin content, and white blood cell (WBC) count.

### Biochemical Investigations

After separation blood plasma was stored at −20°C until use. Next, plasma was subjected to AST, ALP, and ALT analysis using commercial kits (Biomerieux, Marcy l’Etoil, France) according to the manufacturer’s instructions. Furthermore, total protein activity and gamma-glutamyl transferase (GGT) levels in the mouse jejunum were assessed using an approach described by Jollow et al and Buege et al.^25^,^26^

### Histopathology Investigations

Freshly prepared liver samples were embedded in paraffin after fixation in 10% neutral buffered formalin. The sections were then cut into 5 μm thick segments, and hematoxylin and eosin were used for staining according to Avwioro et al.^27^ Liver histology scoring was performed according to the recommendations provided by Ishak et al.^28^

### Expression of Gene

TRIzol (QIAGEN, Hilden, Germany) was utilized to isolate the liver RNA to perform a real-time quantitative polymerase chain reaction (RT-qPCR). The samples were prepared using DNase (Applied Biosystems, Darmstadt, Germany) and transformed into cDNA (QIAGEN, Hilden, Germany) using a reverse transcription kit. SYBR green PCR master mix (QIAGEN, Hilden, Germany) and ABI Prism 7500HT sequence detection equipment (Applied Biosystems, Darmstadt, Germany) were used for PCR. Using primers from Macrogen Inc. (Seoul, South Korea), the levels of interleukin-1b (IL1β), interleukin-10 (IL-10) were measured. The PCR procedure is as follows:^2^ The 2−DDCT approach was used to ascertain the fold change in mRNA expression according to Livak and Schmittgen.^29^

### Statistical Analysis

The differences were analyzed using the one-way analysis of variance (ANOVA) procedure of SPSS Vision 21 statistical software. We applied the Duncan test to compare the mean values of the different compounds. A probability ≤ 0.05 was considered significant (*p*≤0.05). We reported the mean values along with the standard deviation media (SD).

## Results

### Gas Chromatography Mass Spectrometry (GC-MS)

**Figure 1:**
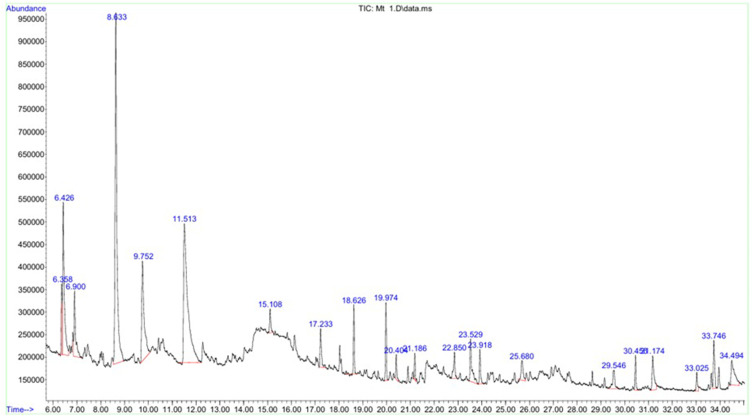
Phytochemical Composition Analysis by GC-MS to C. procera leaf extract.

In recent years, *C. procera*, a plant known for its therapeutic qualities, has garnered significant attention due to its diverse phytochemical profile and potential applications. Table 1 and Figure 1 show the results of a GC-MS analysis detailing the phytochemical composition of a CPLE. Our findings from the analysis show the most abundant compound is Z-3-Methyl-2-hexenoic acid (24.07%), the second compound is 4H-Pyran-4-one, 2.3-dihydro-3,5-dihydroxy-6-methyl- (21.99%), and other significant compounds include 2-Furancarboxaldehyde, 5-methyl- (9.47%) and Furaneol (9.15%).

**Table 1:** Phytochemical Composition Analysis by GC-MS to C. Procera Leaf Extract

Retention Time	Area	Peak Area%	Phytochemicals	MF	Mw
6.358	4,213,944	2.472496	3,4-Furandimethanol	C_6_H_8_O_3_	128
6.426	16,139,147	9.469509	2-Furancarboxaldehyde, 5-methyl-	C_6_H_6_O_2_	110
6.9	6,678,248	3.918406	3-(1-Cyclopentenyl) furan	C_9_H_10_O	134
8.633	41,028,133	24.07291	Z-3-Methyl-2-hexenoic acid	C_7_H_12_O2	128
9.752	15,602,543	9.154661	Furaneol	C_6_H_8_O_3_	128
11.513	37,471,678	21.98619	4H-Pyran-4-one, 2.3-dihydro-3,5-dihydroxy-6-methyl-	C_6_H_8_O_4_	144
15.108	1,668,627	0.979053	1,2,3,4-Tetrahydroisoquinolin-6-ol-1-carboxylic acid	C_10_H_11_NO_3_	193
17.233	3,649,054	2.141052	Phenol, 2-methoxy-5-(1-propenyl)-, (E)-	C_10_H_12_O_2_	164
18.626	4,292,422	2.518542	Naphthalene, 1.2-dihydro-2,5,8-trimethyl-	C_13_H_16_	172
19.974	4,667,309	2.738504	cis-p-Mentha-2,8-dien-1-ol	C_10_H_16O_	152
20.404	1,793,708	1.052443	3-Hydroxy-α-ionene	C_13_H_20_O_2_	208
21.186	1,373,284	0.805763	1-Naphthalenol, 1,2,3,4-tetrahydro-2,5,8-trimethyl-	C_13_H_18_O	190
22.85	1,750,370	1.027015	Cyclopenta[1,3] cyclopropa[1,2]cyclohepten-3(3aH)-one, 1,2,3b,6,7,8-hexahydro-6,6-dimethyl-	C_13_H_18_O	190
23.529	5,111,136	2.998916	4-(3,7,7-Trimethyl-2-oxabicyclo [3.2.0] hept-3-en-1-yl) but-3-en-2-one	C_13_H_18_O_2_	206
23.918	2,556,193	1.499825	Acetic acid, (1,2,3,4,5,6,7,8-octahydro-3,8,8-trimethylnaphth-2-yl) methyl ester	C_16_H_26_O_2_	250
25.68	2,790,292	1.63718	trans-Z-α-Bisabolene epoxide	C_15_H_24_O	220
29.546	1,990,060	1.167651	Z-8-Methyl-9-tetradecenoic acid	C_15_H_28_O_2_	240
30.455	2,195,702	1.28831	Hexadecanoic acid, methyl ester	C_17_H_34_O_2_	270
31.178	3,673,441	2.155361	Estra-1,3,5(10)-trien-17β-ol	C_18_H_24_O	256
33.746	4,221,358	2.476846	7,10,13-Hexadecatrienoic acid, methyl ester	C_17_H_28_O_2_	264
34.494	7,566,133	4.439365	Z, E-2,13-Octadecadien-1-ol	C_18_H_34_O	266

### TPC, TFC, and TTC Measurements

The TPC, TFC, and TTC of the CPLE were estimated (Table 2). The recorded results showed the highest amount of TPC (86.3545 ± 0.585), followed by TFC (30.32 ± 0.205 mg QE/g DW), and TTC (36.04 ± 0.236 mg TAE/g DW).

**Table 2:** CPLE Contains Total Phenols, Flavonoids, and Tannins

Extract	Phenol (mg GAE/g DW)	Flavonoid (mg QE/g DW)	Tannin (mg TAE/g DW)
***C. procera***	86.3545 ± 0.585	30.32 ± 0.205	36.04 ± 0.236

### Antioxidant Activity (DPPH and ABTS)

*C. procera* has antioxidant activity that offers significant protection against various diseases. In the current study, the 2.2-diphenyl-1-picrylhydrazyl (DPPH) assay was used to determine the antioxidant activity of CPLE. Increasing the concentration of leaf extract increased the scavenging ability of DPPH radicals, as demonstrated by the results. The rate of (DPPH and ABTS) at 1000 was (301.6165 ± 403.212) and (319.1692 ± 393.078), respectively. (Table 3). The radical-scavenging capabilities of phenolic components are primarily responsible for the antioxidant effects of plant products. Based on the DPPH and ABTS assays, the IC50 values for the CPLE were (525.7179 ± 42.6753 μg/mL and 107.5536 ± 0.1274 μg/mL), respectively.

**Table 3:** Antioxidant Activity of Concentrations with Offline DPPH and ABTS IC_50_ assay of CPLE

Concentrations	Inhibition%	
	DPPH	ABTS
31.25	7.4720± 0.5458	21.5554 ± 4.199
62.5	9.4823 ± 0.3371	35.4336 ± 11.721
125	18.1031 ± 0.3253	55.2176 ± 30.218
250	28.3049 ± 0.4179	72.9355 ± 76.675
500	49.71261 ± 0.1016	83.3672 ± 180.408
1000	68.8221 ± 0.7139	92.2256 ± 393.078
IC_50_ (*μ*g/mL)	525.7179 ± 42.6753	107.5536 ± 0.1274

### AgNP Biosynthesis and Characterization

**Figure 2:**
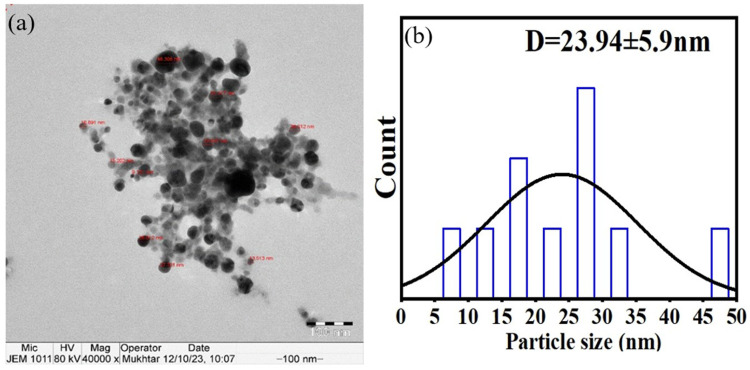
The CPLE AgNPs characterization, (a) TEM image of CPLE AgNPs. (b) the histogram distribution of particle size of CPLE AgNPs. The scale bar is 100 nm.

After processing, the leaf extracts were combined with AgNPs. The CPLE AgNPs were examined using Transmission Electron Microscopy (TEM) at room 22°C. Examination showed that the nanoparticles were spherical on a smooth surface with a spherical morphology, and their size ranged from 9 to 48 nm (Figure 2). Characterization of CPLE AgNPs, as shown in transmission electron micrographs. Additionally, the image demonstrates that there are no plant extract residues left in the final product, indicating a highly pure and morphologically well-produced nanostructure. Figures a and b depict the TEM picture and histogram distribution of particle size for CPLE AgNPs. As demonstrated in Figure (1a), the CPLE-mediated synthesis of AgNPs produced spherical nanoparticles with homogeneous sizes. (Figure 1) shows that the average size of CPLE AgNPs is 23.94±5.9 nm.

### UV-Vis Analysis

**Figure 3:**
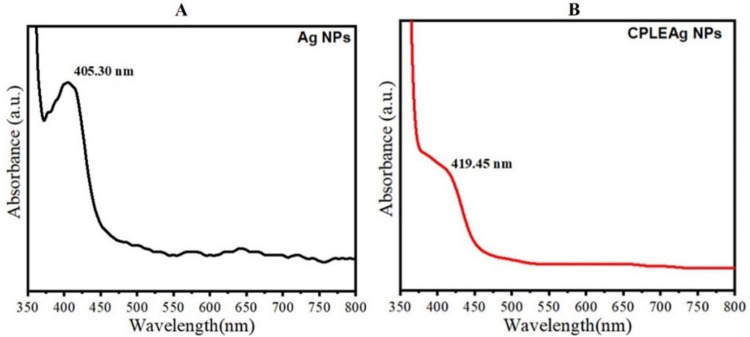
(**A**) UV-vis absorption of AgNPs and (**B**) biosynthesized of CPLE AgNPs.

The UV-vis absorption spectra of the prepared AgNPs and CPLE are presented in Figures 3A and B, respectively. It can be observed that the vis absorption peaks of prepared Ag NPs and CPLE AgNPs were 405.30 nm and 419.45 nm, respectively. This shift in the peaks indicated that the use of *C. procera* improved the optical properties of the synthesized AgNPs. The UV results suggest that these prepared samples can be used for environmental remediation and medical applications (Figure 3).

### Parasitemia%

On day 7 p.i., parasitemia peaked at approximately 42%. Infected mice were treated with CPLE AgNPs on days 3, 4, 5, 6, and 7 post-infection, and the parasitemia decreased by approximately 42.4 ± 15.7, 87.1 ± 0.8, 94.6 ± 4, 97.8 ± 1.4, and 98.5 ± 1.2%, respectively (Table 4).

**Table 4:** Impact of CPLE AgNPs on the Inhibition of Parasitemia in Mice Carrying P. Berghei-Infection

Group	Suppression (%)				
	Day 3	Day 4	Day 5	Day 6	Day 7
**Infected**	0	0	0	0	0
**Infected + 50 mg/kg CPLE AgNPs**	42.4 ± 15.7	87.1 ± 0.8	94.6 ± 4	97.8 ± 1.4	98.5 ± 1.2

**Figure 4:**
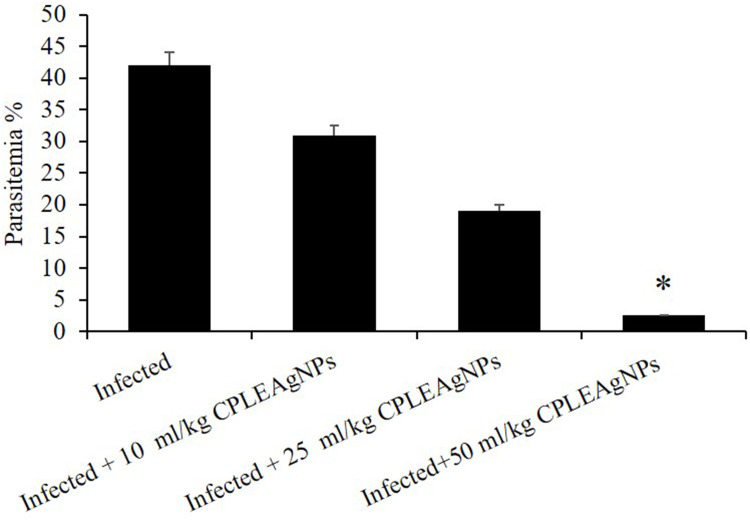
In mice infected with *P. berghei*, CPLE AgNPs reduced parasitemia. (*) Significant at *p* < 0.01 compared to the infected group on day 7 parasitemia.

**Figure 5:**
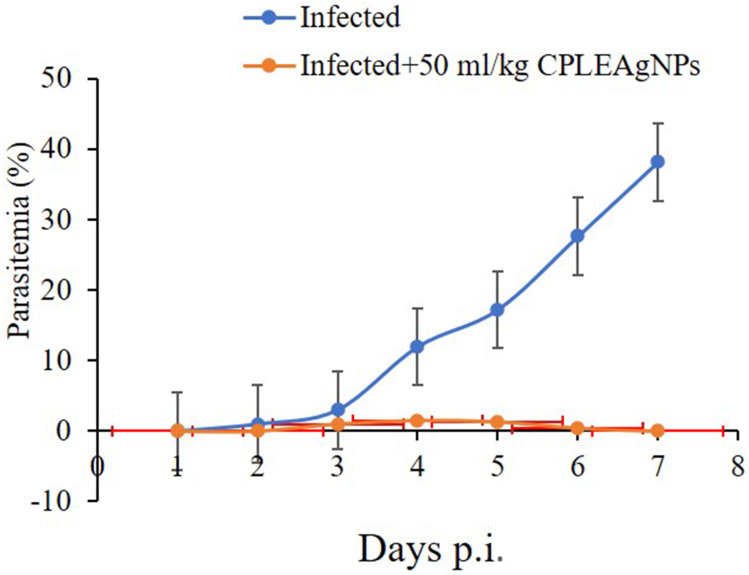
*C. procera-*induced parasitemia changes in mice due to infection with *P. berghei* on day 7 p.i.

The dose of CPLE AgNPs used in subsequent testing was 50 mg/kg (Figure 4). This choice was based on the parasitemia statistics presented in Figure 5. On day 7 post-infection, the parasitemia level in the infected group was approximately 42%. The group treated with 50 mg/kg CPLE AgNPs showed < 3% parasitemia (Figure 5).

**Figure 6:**
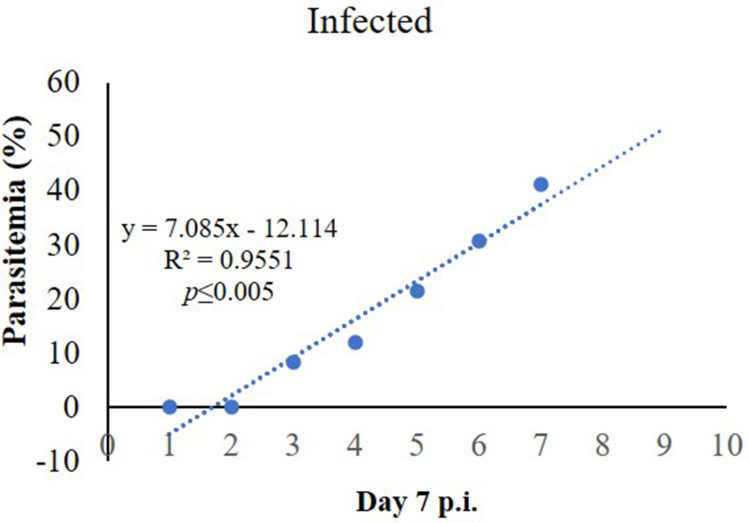
Liner correlation between parasitemia % of *P. berghei*-Infected group and number of days until 7 days.

The CPLE induced a marked reduction in parasitemia from day 4 after infection with *P. berghei* infected with erythrocytes until I arrived on the seventh day at 39.5% relative to the infected mice (Figure 6).

### Liver Index

**Figure 7:**
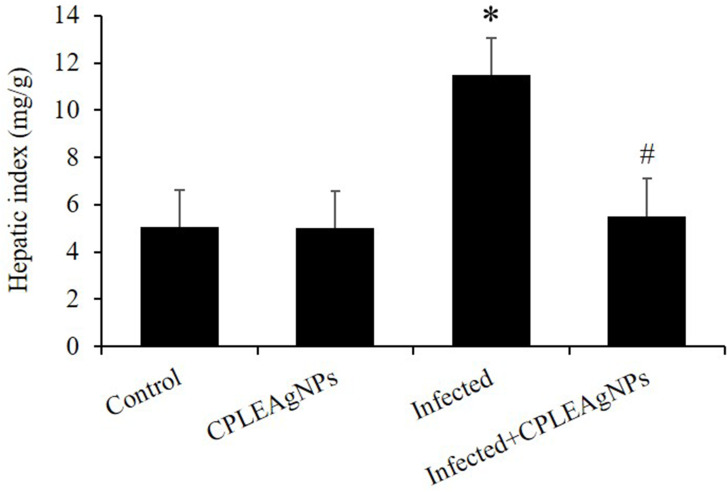
The liver index of mice infected with *P. berghei* was altered as a result of the administration of CPLE AgNPs.In comparison to the control group and the infected group, the symbols (*) and (#) are statistically significant at a level of *p* < 0.01.

The infection caused hepatomegaly, which was observed in larger livers of mice on day 7 after infection with *P. berghei* (Figure 7). CPLE AgNPs significantly reduced the liver index by approximately two to three times that of the affected group.

### Hematological Studies (WBCs)

On day 7 p.i., *P. berghei* infection in mice reduced the number of WBCs; hematological parameters were restored after treatment with CPLE AgNPs (Table 4).

P. *berghei* infection in mice caused a mark in leucocytes of 8.8×109 ±3.4 /L. Treatment with CPLE AgNPs on day 7 p.i. These resulted in a higher leucocyte count (2.4×109±0.7/L) compared to the infected group (Table 5). Mice infected with *P. berghei* had higher levels of basophils (5.75±3.6), neutrophils (10±2.3), eosin (12±5.38), and lymphocytes (12.75±3.9), whereas white monocytes (1.8±0.81) were unchanged compared to the control group. These parameters changed when infected mice were treated with CPLE AgNPs.

**Table 5:** Changes in the Number of White Blood Cells (WBCs) in the Blood of P. Berghei-Infected Mice Before and After Treatment with CPLE AgNPs

Group	WBC (×10^9^/L)	Neutrophil	Basophil	Eosinophil	Monocyte	lymphocyte
**Control**	3.4±0.4	7.25±3.3	2.4±1.7	2.8±1.3	1.8±0.84	11±1.8
**CPLE AgNPs**	1.96±0.9*	6.4±1.8	6.5±3.08	6.4±2.4	2.8±1.3	12.4±5.02
**Infected**	8.8±3.9*	10±2.3	5.75±3.6	12±5.38	1.8±0.81	12.75±3.9
**Infected +** **CPLE AgNPs**	2.4±0.7^*#^	9±3.51	3.8±3.11	9.2±4.32	3.5±1.3	9.5±4.4

**Notes**: Values are mean ± SD. (*) is significant at *p* < 0.01 compared the non-infected groups and (#) is significant at *p* < 0.01 compared infected groups.

### Impact of Treatment of CPLE AgNP_S_ on the Infected Liver Cells

**Figure 8:**
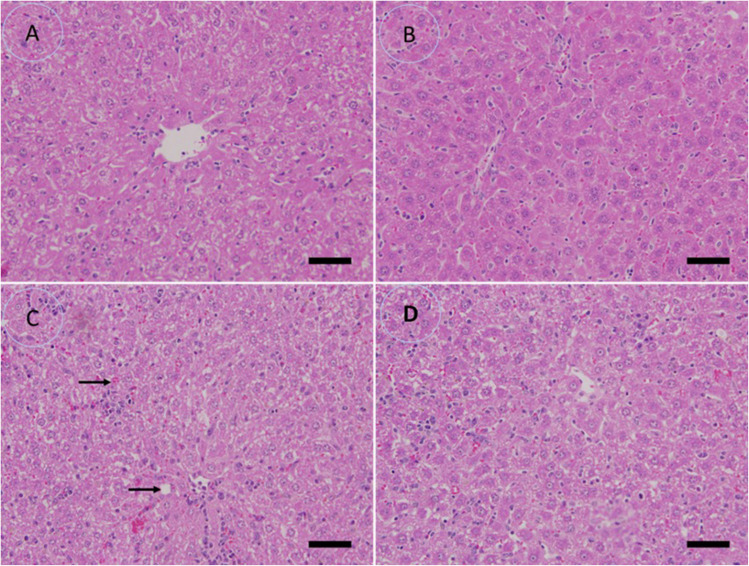
The study examined the effects of CPLE on the liver histopathology of *P. berghei*-infected mice. (**A**) non-infected group. (**B**) CPLE AgNPs-infected group. (**C**) An infected group with inflammation, malaria pigment (arrow), and hepatocytic vacuolation (arrowhead). (**D**) Infected + 50 mL/kg CPLE AgNPs group with improved structure. Scale bar = 10 μm.

Mice infected with *P. berghei* showed severe histological changes in the liver, along with extensive damage from inflammatory cells. Hepatic infiltration, the appearance of apoptotic bodies, dilated sinusoids, malaria pigments, and hyperplasia of Kupffer cells are manifestations of this condition. In contrast, the liver architecture of the CPLE AgNP-treated and control groups was normal, which reduced the aforementioned histological abnormalities in the liver. In mice treated with CPLE AgNPs, hepatic lesions were reduced and the liver score decreased, which was associated with a decrease in inflammatory cell infiltration (Figure 8).

**Figure 9:**
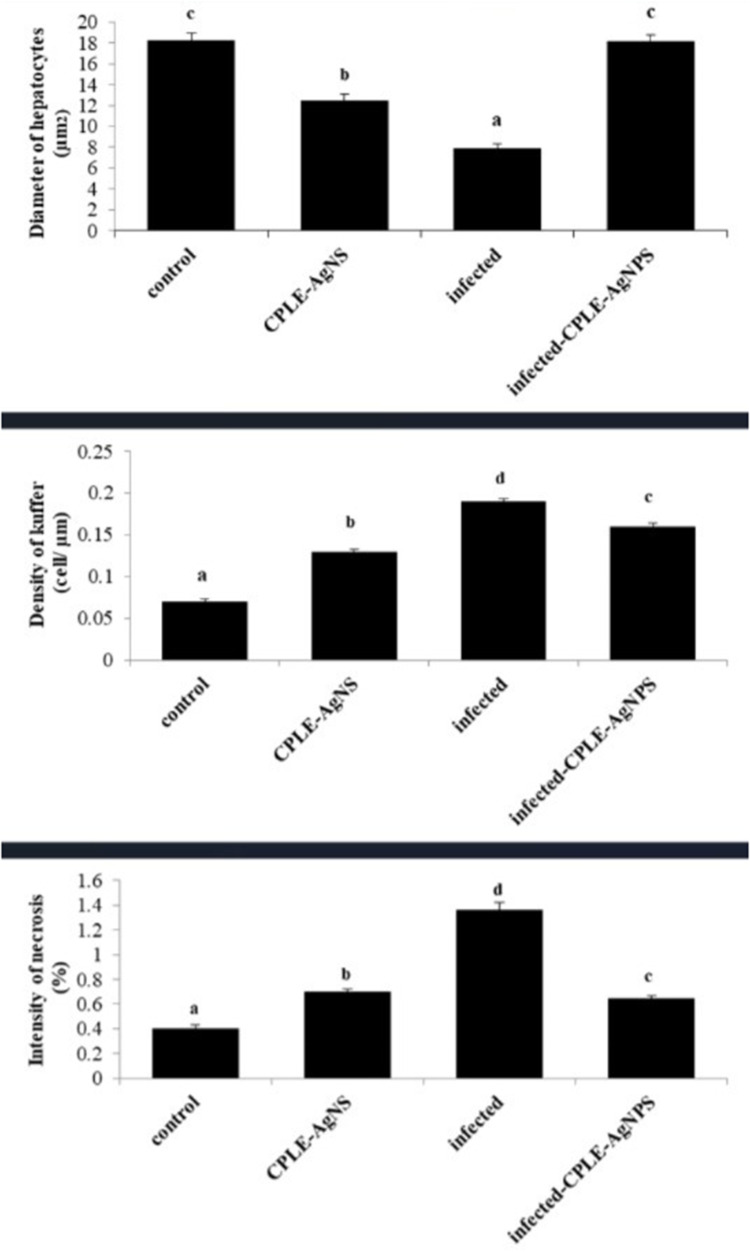
Bar charts showing the morphometric analysis of histological parameters of liver tissue which (**A**) Hepatocytes diameter, (**B**) density of Kupffer cell, and (**C**) necrosis intensity, in four groups: Control; CPLE AgNPS group; infected group, and treated with CPLE AgNPS group. Values are represented as Mean ± STDV & n = 10 animals. Means within the same parameter and not sharing a common superscript symbol(s) differ significantly at *p* < 0.05, and values that are recorded with non-significance difference (n.s).

**Figure 10:**
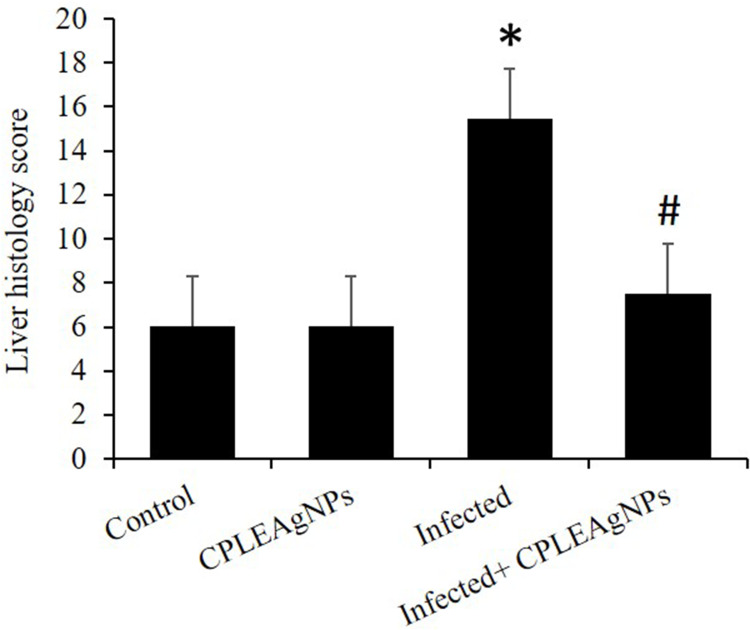
CPLE AgNPs induced changes in the liver histology score of mice infected with *P. berghei*. (*) and (#) are significant at *p* < 0.01 against control and infected groups, respectively.

The morphometric analysis expressed a little shrinkage (12.48±0.61 µm2) in the infected CPLE-AgNP_S_ hepatocellular cells significantly (*p*<0.05) is comparable to normal hepatocytes (18.22±0.72 µm2). On the contrary, the density of Kupffer cells (0.13±0.00 cell/ µm2), and necrotic cells (0.70±0.02%) exhibited an elevated intensity (*p*<0.05) in the infected CPLE AgNPS hepatic tissue in comparison with the healthy tissues. While the infected hepatocytes showed severe (*p*<0.001) reduction in their size (7.93±0.40 µm2) more than the control group. Also, the Kupffer cell density (0.19±0.003 cell/ µm2) and necrosis intensity (1.36±0.06%) were higher significantly (*p*<0.001) than the healthy ones. On the other hand, the infected tissue that was treated with CPLE AgNP_S_ exhibited a rapid nonsignificant (*p*>0.05) recovery to the normal diameter in their hepatocytes size (18.19±0.55 µm2), and also well regression in both Kupffer (0.16±0.004 cell/ µm2) and necrotic cells to the normal levels as illustrated (Figure 9). The liver activity index of the infected group ranged from 14 to 16 according to Ishak’s score. The liver index decreased to 5–7 in mice (Figure 10).

### Biochemical Investigations

**Figure 11:**
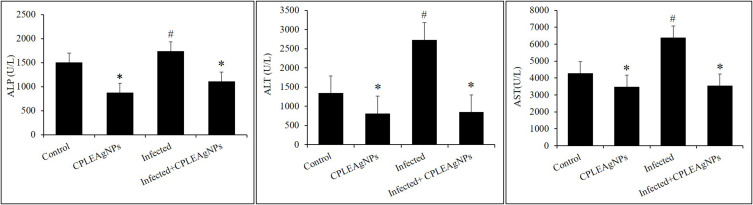
Impact of CPLE AgNPs on liver functions. a: ALP, b: AST, and c: ALT in the liver of *P. berghei*-infected mice. (*) significant differences at *p* < 0.001, and # at *p* < 0.05. compared to the control.

**Figure 12:**
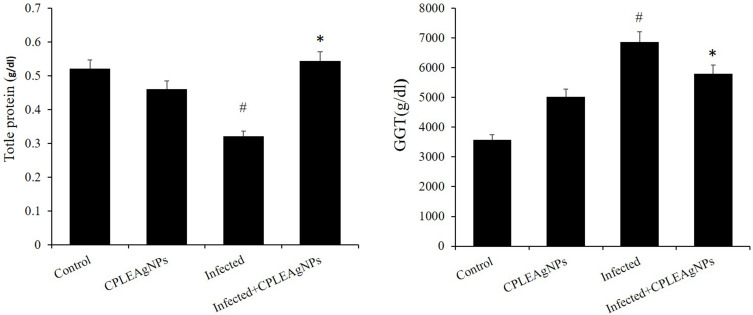
The levels of oxidative stress indicators (TP) in the jejunum of mice infected with *P. berghei* are influenced by CPLE AgNPs. Every value is displayed as the mean ± SE. * Significant change (*p* < 0.05) from the control group. # Significant change (*p*<0.05) from the infected group.

The livers of the infected group showed significant dysfunction (*p* < 0.01). Compared to the non-infected group, the blood plasma ALP, ALT, and AST levels increased to 67 ± 7, 66 ± 5, and 318 ± 10 U/L, respectively. The administration of CPLE AgNPs to mice resulted in a decrease in ALP, AST, and ALT activities (Figure 11). The infected group exhibited significantly higher liver GGT levels and lower TP levels than those in the control group (Figure 12).

### Expression of Gene

**Figure 13:**
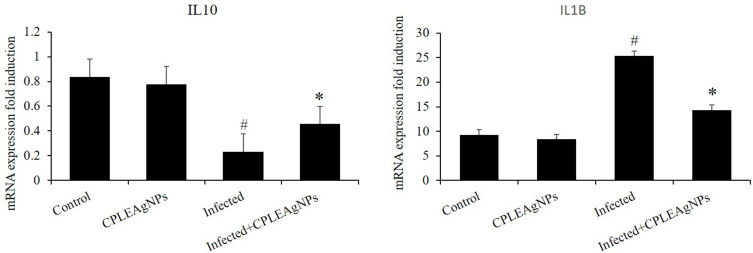
CPLE AgNPs influence the expression of IL-10 and IL-2 in the liver of mice infected with *P. berghei*. Data are presented as mean ± SD at *p* ≤ 0.001. * and #: significance against the control and infected groups, respectively.

Infection with *P. berghei* caused liver tissue inflammation, which was shown by a significant increase (*p* < 0.05) in the levels of the pro-inflammatory cytokines IL-10 and IL-1β (Figure 13). However, administration of CPLE AgNPs significantly suppressed this inflammatory response, demonstrating the protective effect of CPLE AgNPs against inflammatory events triggered during malaria development.

## Discussion

Most antimalarial medications currently in use have been rendered ineffective because of drug resistance; therefore, there is an urgent and ongoing need to find new antimalarial medicines.^30^ Natural products are essential in the search for novel therapeutic targets for human illnesses. Consequently, society is placing increasing pressure on the development of new technologies, pushing for advancements in clean and green technology. The best way to fight different parasitic illnesses is through nanotechnology.^31^ According to Ullah Khan et al,^32^ the most economical, environmentally friendly, and efficient method for producing AgNPs is plant synthesis.

*C. procera* is regarded as a source of biologically active substances that are high in phenolics, flavonoids, and tannins because its major constituents are polar and highly soluble in methanol. This is similar to the findings in the literature.^33^,^34^ These chemicals may contribute to the antimalarial action and lower parasitemia observed after treating infected animals. Ahmad et al^35^ recently reported that *C. procera* methanolic leaf extracts used against antimicrobials and *P. berghei* significantly decreased malarial-induced physiological imbalances in liver and kidney biomarkers, as well as serum electrolytes, owing to the presence of phytochemically active compounds in the extract that induce malaria and liver and renal dysfunction.^36^ A study by Al-Rowaily et al^37^ reported that *C. procera* extract contains a high concentration of carotenoids.^37^ A redox process that releases free radicals, such as hydroxyl radicals, hydrogen peroxide, and superoxide anions, occurs when cells use oxygen to create energy.^38^ The antioxidant properties of plant extracts were evaluated using various indices in several model systems to ensure their effectiveness. In this study, the *C. procera* extract demonstrated significant antioxidant activity. Samani et al achieved 63.69 and 64.33% free radical inhibition using DPPH and ABTS, respectively, at a concentration of 1000 ppm of thyme essential oil.^39^ This difference could be due to the extraction method and extraction of the extract from different parts of the plant and the extraction method.^40^ Secondary metabolites, particularly plant phenols and carotenoids, are a large group of compounds that act as primary antioxidants.^17^ Generally, the amount of antioxidant compounds in a plant determines its antioxidant activity; plants with more phenolic compounds exhibit higher antioxidant activity.^41^ Additionally, antioxidant activity increased with increasing sample concentrations, which is consistent with the findings of Ismail and Hong.^42^ Plants produce synthetic AgNPs, which vary widely in size and shape.^43^ The synthesized AgNPs were largely spherical, TEM image were excellent in agreement with earlier studies.^2^ Recently, researchers have studied the hepatoprotective and antiplasmodial characteristics of AgNPs in male C57BL/6 mice.^44^ Researchers have used the hepatic tissue of female mice to demonstrate the effectiveness of CPLE AgNPs in controlling the liver status after *P. berghei* infection. A study by Mehlhorn,^31^ used *P. berghei* as a mouse parasite model to study the role of the liver during infection because it shares many traits with *P. falciparum*, a human malaria parasite. This study found that CPLEAgNPs reduced parasitemia in infected mice in a manner similar to that observed with medicine-manufactured drugs. The presence of numerous predicted components in the utilized CPLE AgNPs may allow specialists to identify and exploit these compounds for antiplasmodial activity.^45^ It has been confirmed that silver has been utilized since ancient times to treat a variety of microbiological illnesses.

A study by Murshed et al^2^ showed that administering CPLE AgNPs to *P. berghei*-infected male mice reduced parasitemia. This was attributed to the plant extract, which contained the antimalarial chemical 2.6-di-t-butyl-p-benzoquinone.^46^ In this study, CPLE AgNPs drastically reduced parasitemia in infected female mice. This is attributable to the action.^47^

The possible therapeutic effects of the extracts on liver enzyme activity could be related to parasite eradication and/or antioxidant effects arising from their bioactive ingredients.^48^,^49^

The decrease in erythrocyte count and hemoglobin concentration in *P. berghei*-infected mice suggests that they were anemic.^44^ Anemia alters tissue physiology and histology induces oxidative damage, and produces free radicals. A previous study Omodeo-Sale et al^50^ found that oxidative damage enhances the development of anemia during *P. falciparum* infection. This is due to an imbalance between oxidants and antioxidants, which produce free radicals that induce oxidative injury in the liver.^51^ Furthermore, anemia is caused by parasites using hemoglobin as a source of sustenance, releasing haem, which promotes oxidative damage and causes histological alterations in the liver.^52^ These biochemical and histological findings are similar to recent studies^2^ on the protective effects of herbal extracts on the spleen against *P. chabaudi* infection.

Our data suggested that *P. berghei*-induced hepatic inflammation is accompanied by an inflammatory response in the liver. Furthermore, our findings show that CPLE AgNPs kill Plasmodium parasites in mice while also having anti-inflammatory properties that preserve the liver.

Infection with *P. berghei* may cause an inflammatory reaction in the mouse liver. The reaction involves changes in liver structure, Tp and GGT, and inflammatory cytokines IL-1β, and IL-10. Plasmodium infection is distinguished by early and intensive cytokine-mediated effector mechanisms that kill or destroy parasite-infected cells and are associated with both acquired and innate immune responses.^53^ Hyperparasitemia has been linked to elevated levels of IL-1β, and IL-10.^54^ Furthermore, inflamed liver cells, activated leukocytes, and macrophages produce high levels of pro-inflammatory cytokines and enzymes during inflammation, which aids in the initiation of the innate immune response.^55^ Interestingly, CPLE AgNPs treatment reduced the development of inflammatory reactions following *P. berghei* infection by downregulating cytokines and reducing oxidative alterations in the liver. All CPLE AgNPs-mediated lower inflammatory responses were initially attributable to a decrease in the number of parasitized erythrocytes. Several studies support our finding that medicinal plants can reduce parasitemia and protect the liver from inflammation.^55^

We propose that CPLE AgNPs are capable of preserving hepatic tissue following malaria-induced infection, as indicated by increased parasitemia and histological structure. Furthermore, CPLE AgNPs restored the equilibrium of oxidants and antioxidants, and decreased inflammation in the liver. These data suggested that CPLE AgNPs are promising hepatoprotective drugs against malaria. Further research is needed to understand the processes underlying the ability of the liver to respond to both *P. berghei* and CPLE AgNPs.

## Conclusions

Recent investigations have demonstrated the potential use of CPLE AgNPs as antimalarial drugs in the therapeutic domain. The results showed that CPLE AgNPs provided antioxidant and anti-inflammatory protection against *P. berghei*-induced liver damage, leading to a significant improvement in the histological alterations in the livers of mice. To develop important therapeutic drugs based on the active phytochemical constituents of CPLE AgNPs, further research is necessary to isolate and test the activity of each compound, as well as to uncover the plant’s additional pharmacological and therapeutic potential.
